# Human Papillomavirus-Associated Tumor Extracellular Vesicles in HPV^+^ Tumor Microenvironments

**DOI:** 10.3390/jcm12175668

**Published:** 2023-08-31

**Authors:** Steven F. Gameiro, Kaitlyn M. Flondra

**Affiliations:** 1 McMaster Immunology Research Centre, Department of Medicine, McMaster University, Hamilton, ON L8S 4K1, Canada; 2 Department of Anatomy and Cell Biology, Schulich School of Medicine and Dentistry, London, ON N6A 5C1, Canada; kflondra@uwo.ca

**Keywords:** exosomes, microvesicles, head and neck cancer, cervical cancer, TME, biomarkers, liquid biopsy

## Abstract

Most infections with human papillomaviruses (HPVs) are self-resolving and asymptomatic. However, some infections can lead to the development of cancer at different mucosal sites, such as the cervix and the head and neck. Head and neck cancers (HNCs) are dichotomized into HPV-positive (HPV^+^) or HPV-negative (HPV^−^) based on their respective etiologies. Notably, the tumor microenvironment (TME) of the HPV^+^ subtype has an immune landscape characterized with increased immune infiltration, higher levels of T cell activation, and higher levels of immunoregulatory stimuli compared to their HPV^−^ counterparts. Both enveloped and nonenveloped viruses hijack the extracellular vesicle (EV) biogenesis pathway to deploy a “trojan horse” strategy with a pseudoviral envelope to enhance infectivity and evade inflammation. EVs derived from HPV-infected tumor cells could allow for the stealth transport of viral cargo to neighboring nonmalignant cellular populations or infiltrating immune cells within the TME. Furthermore, viral cargo or altered cellular cargo from HPV-associated tumor EVs (HPV-TEVs) could alter the functional state or biological responses of the recipient cellular populations, which could shape the distinctive HPV^+^ TME. This review will cover the impact of EVs released from HPV-infected cells on HPV-induced carcinogenesis, their role in shaping the distinctive HPV^+^ tumor microenvironment, and current efforts to develop a painless EV-based liquid biopsy for HPV^+^ cancers.

## 1. Introduction

To date, there have been over 400 human papillomavirus (HPV) types identified, all with exclusive tropism for either cutaneous or mucosal epithelia [1]. Twelve mucosa-associated HPV types (HPV16, 18, 31, 33, 35, 39, 45, 51, 52, 56, 58, and 59) have been designated as potent biological carcinogens by the World Health Organization’s (WHOs) International Agency for Research on Cancer (IARC) [2]. These types are commonly grouped as high-risk (HR) HPV types due to their carcinogenic potential. Indeed, HR HPVs have been estimated to cause approximately 4.1% of the global cancer burden, according to the latest GloboCAN estimates from 2018 [3]. Mucosa-associated HPVs are a common sexually transmitted virus, with most infections being self-resolving and asymptomatic [4]. However, some infections, particularly with HR HPV types, can potentially lead to the development of cancer at different mucosal sites, such as the cervix, vagina, vulva, anus, penis, and the head and neck region (Table 1) [3,5].

The number of new cases of head and neck cancers (HNCs) caused by HR HPVs are on the rise with the concomitant decline in new cases caused by non-viral factors such as smoking and excessive alcohol consumption [6,7]. HNCs are dichotomized based on their distinct etiologies: HPV-positive (HPV^+^) or HPV-negative (HPV^−^) [5,8,9]. A number of studies have revealed that the tumor microenvironment (TME) of the HPV^+^ subtype has an immune landscape characterized with increased immune infiltration, higher levels of T cell activation, and higher levels of immunoregulatory stimuli compared to their HPV^−^ counterparts [5,10,11,12,13,14,15,16]. In addition, there is evidence supporting the same immunological phenomena observed in HNCs in HPV^+^ cervical cancers (CCs) [17,18]; however, due to virtually all CCs being caused by HPV, this area is currently understudied because it lacks the benefit of an anatomically similar HPV^−^ tumor control.

All cells, regardless of their respective domains, release extracellular vesicles (EVs) during normal physiological functions and in diseased states [19]. The molecular cargo of EVs is reflective of their cell of origin (COO) as they contain similar constituents to those of the originating cell [20]. This can include DNA, RNA (coding and noncoding), lipids, metabolites, and different types of proteins (transmembrane, cytosolic, and nuclear) [21,22,23,24]. EVs play a role in the maintenance of homeostasis through the removal of cellular waste products and in the dynamic intercellular communication network [25]. The molecular cargo (nucleic acids, proteins, and metabolites) delivered by EVs to recipient cells can effectively alter their functional state and biological responses [20]. Due to the inherent heterogeneity of EVs, which stems from their COO-dictated molecular cargo, their effect on recipient cells is functionally variable. Indeed, in different target cell types, EVs have been shown to impact immune responses, viral pathogenicity, carcinogenesis, and the evolution of the TME [20]. Furthermore, the intrinsic properties of EVs and their ability to influence complex cellular systems have led to their advancement as non-immunogenic delivery systems to control various diseases, including infectious diseases, neurodegenerative disorders, and cancers. EVs can be reprogrammed to deliver therapeutic cargos, such as drugs, immunogenic peptides, or functional enzymes to a desired cellular target [26]. In addition, EVs are being advanced as potential diagnostic and/or prognostic liquid-based biomarkers of disease burden [27]. Their presence in all biological fluids, ease of sampling (liquid biopsies), and multicomponent cargo—reflective of their COO—can aid in unravelling disease progression and/or response to therapy.

The distinct immunological phenomena observed in HPV-induced TMEs could be due to the continuous expression of the *E6* and *E7* oncogenes within HPV-infected tumor cells [16,28,29,30,31,32]. The existence of foreign viral proteins within infected cells could trigger the recruitment of the observed tumor-infiltrating immune cells—seemingly primed for antitumor activity. Nevertheless, in the absence of an active or productive HPV infection, there is no clearance of these HPV-infected tumor cells nor resolution of the TME. Therefore, an alternative mechanism could explain the observed phenomena of immune mobilization into the HPV^+^ TME and lack of tumor eradication. There is evidence that HPV viral oncogenes *E6* and *E7* are found in EVs regardless of the integration status of the viral genome within the infected cell [33]. Furthermore, other studies have highlighted that EVs from HPV-infected tumor cells contain other HPV-associated viral cargo or altered molecular cargo of cellular origin [33,34,35,36]. A proposed advantage of viruses using EVs is to evade antiviral immunity while promoting viral infection through the dissemination of viral components [37]. Indeed, it has been documented that both enveloped and nonenveloped viruses hijack the EV biogenesis pathway to deploy a “trojan horse” strategy with a pseudoviral envelope to enhance infectivity and evade inflammation [38,39,40,41,42].

EVs derived from HPV-infected tumor cells could allow for the stealth transport of viral cargo to neighboring nonmalignant cellular populations or infiltrating immune cells within the TME. Furthermore, viral cargo or altered cellular cargo from HPV-associated tumor EVs (HPV-TEVs) could alter the functional state or biological responses of the recipient cellular populations, which could contribute to the virally induced, immune-inflamed, HPV^+^ TME. These potential HPV-driven biological modifications mediated by HPV-TEVs could influence tumor survival, invasiveness, metastatic dissemination, or patient outcomes and therapeutic responses. Unraveling the role of HPV-TEVs in the TME has the potential to help guide the rational design of therapies that can modulate and/or mobilize the local immune ecosystem towards an antitumor phenotype. In addition, the in-depth characterization of the multicomponent cargo of HPV-TEVs could be used in the development of EV-based liquid biopsies that are painless and could provide “real-time” insights into disease burden.

In this review, we will provide a brief primer on the biology of EVs, examine the current evidence of EVs released from nonmalignant and malignant HPV-infected cells, their impact on HPV-induced carcinogenesis, and their role in shaping the distinctive HPV^+^ tumor microenvironment. In addition, we will discuss current efforts to develop an EV-based liquid biopsy for HPV^+^ cancers for diagnostic and/or prognostic purposes.

## 2. The Biology of Extracellular Vesicles

EVs are defined as membrane vesicles secreted by cells into the extracellular space that contain cytosol and molecular components from the secreting cells enclosed in a lipid bilayer [43]. This process is conserved throughout evolution with all cells, regardless of their respective domains (i.e., Eukarya, Bacteria, and Archaea), releasing vesicles into the extracellular environment during normal physiological functions and in diseased states [19,43,44]. In multicellular organisms, such as mammals, EVs have been isolated from diverse bodily fluids that include urine, semen, blood, saliva, ascites, bile, cerebrospinal fluid, amniotic fluid, breast milk, and cervical–vaginal fluid. EV is an umbrella term that encompasses a diverse number of different types of vesicles that are released into the extracellular space. In the literature, different types of EVs have been named according to their size (e.g., microvesicles or nanovesicles), their cell/tissue of origin (e.g., oncosomes or tumor EVs), or their presence in the extracellular space (e.g., exosomes or ectosomes) [43].

**Figure 1:**
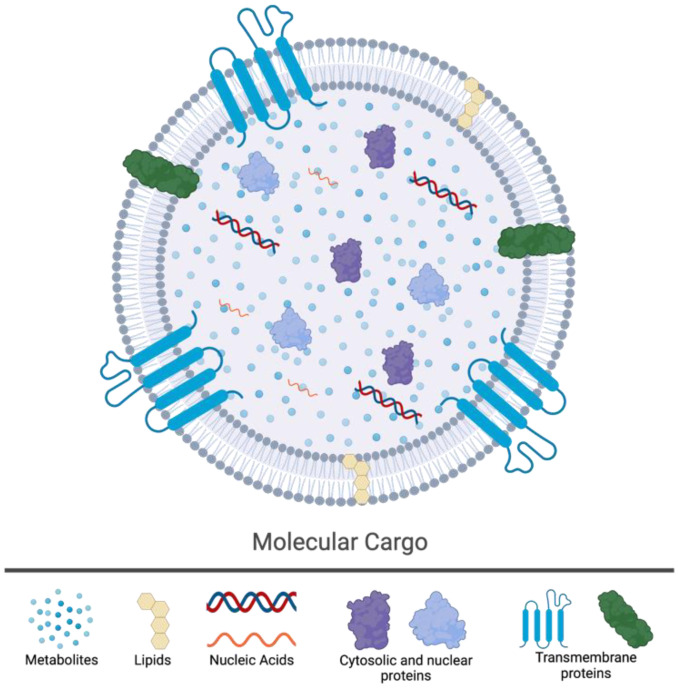
Schematic of an extracellular vesicle with common molecular cargo. All cells release extracellular vesicles (EVs) during normal physiological functions and in diseased states. EVs play an important role in the maintenance of homeostasis and in cell-to-cell communication. The molecular cargo of EVs commonly include metabolites, lipids, nucleic acids (DNA and RNA), proteins of cytosolic or nuclear origin, and transmembrane proteins. Created with BioRender.com (accessed on 6 July 2023).

**Figure 2:**
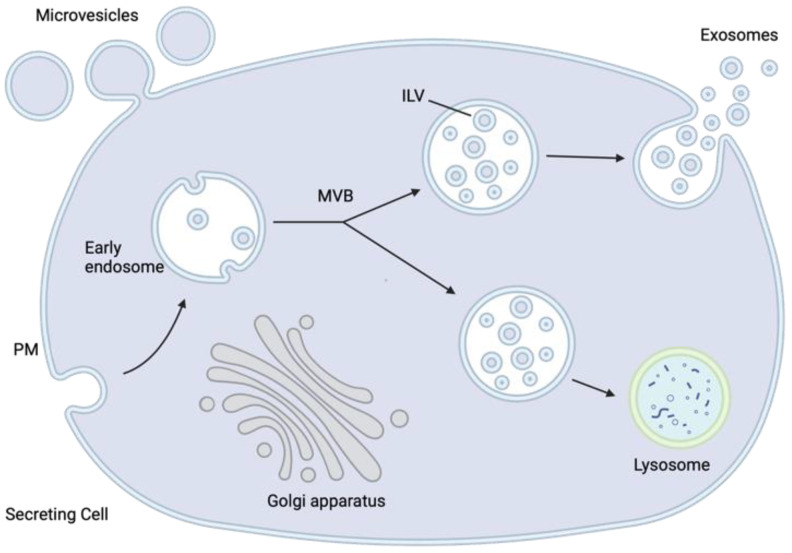
Schematic representation of the different types of extracellular vesicles released by eukaryotic cells. Exosomes are released by a cell into the extracellular space following fusion between the intraluminal vesicle (ILV)-containing multivesicular bodies (MVBs) with the plasma membrane (PM). Microvesicles are formed by the outward budding of the PM and subsequent release into the extracellular environment. Created with BioRender.com (accessed on 17 August 2023).

**Figure 3:**
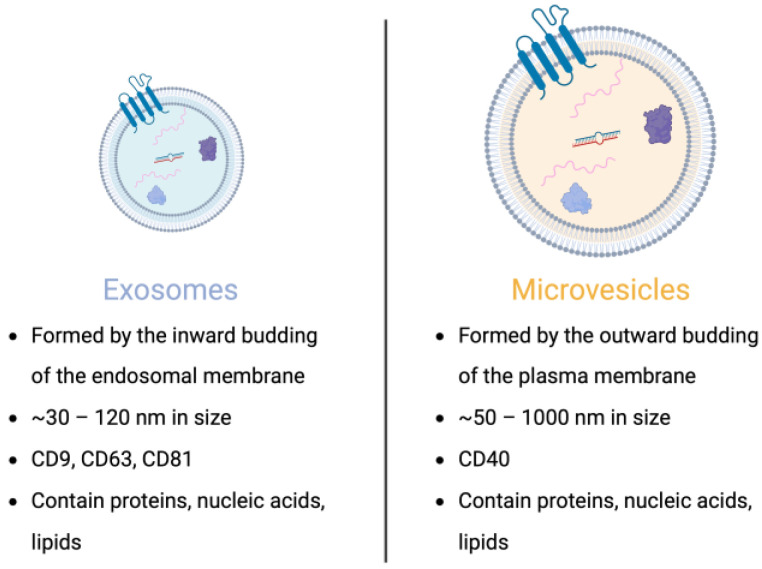
Extracellular vesicle subtypes. There are 2 major subtypes of extracellular vesicles: exosomes and microvesicles. They are differentiated by their size, the manner by which they are formed, and the proteins that they are enriched with. Created with BioRender.com (accessed on 17 August 2023).

In the early 1980s, studies examining the fate of the transferrin glycoprotein in maturing reticulocytes—immature red blood cells—identified a pathway by which an intermediate multivesicular body (MVB) containing multiple intraluminal vesicles (ILVs) fuses with the cell’s plasma membrane, releasing the ILVs into the extracellular space [43,45]. These EVs, once thought of as simply vessels of cellular waste, have been found to be highly diverse in their contents and purpose, containing multitudes of molecules that are representative of their COO. Their molecular cargo can include DNA, RNA (coding and noncoding), lipids, metabolites, and different types of proteins (trans-membrane, cytosolic, and nuclear) (Figure 1) [21,22,23,24]. EVs released by a cell into the extracellular environment as a consequence of the fusion between MVBs and the plasma membrane—liberating the ILVs within—are defined as exosomes (Figure 2). Furthermore, exosomes can be characterized by size (~30–120 nm) and protein biomarkers (e.g., CD9, CD63, and CD81). It is important to note that exosomes are only one type of EV and are distinct from others such as microvesicles (MVs) (Figure 3). The EVs that result from the fusion of MVBs with the plasma membrane are considered exosomes, whereas MVs are both formed and released at the plasma membrane (Figure 2) [46].

EV biogenesis, specifically those of exosomes, begins with the maturation of the early endosome into late endosomes/MVBs. Once MVBs have been formed, they are mobilized and directed to the plasma membrane or the lysosome, where they may fuse membranes and release their ILVs into the extracellular space or into the lysosome for degradation, respectively. Notably, soluble N-ethylmaleimide-sensitive factor attachment protein receptors (SNAREs) appear critical in mediating MVB fusion with the plasma membrane; however, very little is understood about the regulation of the balance between EV secretion or their targeting to the lysosome for degradation [47]. The formation of MVBs from early endosomes is regulated by the endosomal sorting complex required for transport (ESCRT) protein complexes, ESCRT 0, I, II, and III, as well as associated ATPase Vps4 [48]. Cellular contents that are to be packaged into EVs must first be ubiquitinated. Ubiquitinated compounds interact with ESCRT 0 through a ubiquitin-binding domain and are sequestered in clusters. A clathrin-binding motif localizes ESCRT 0 to the membrane of the forming endosome. ILV formation is the following step of endosome maturation and occurs through budding of the endosomal membrane, induced by ESCRT I and II. Budding ILV precursors are separated from the endosomal membrane through ESCRT III [49].

Molecular mechanisms that govern MVB targeting and fusion to the plasma membrane remain unclear, as is the regulation of balance between secretory and degradation pathways. Not every MVB is fated for secretion, and it appears that molecular tagging of an MVB-limiting membrane-associated protein, TSG101, tips the balance towards degradation. Indeed, it has been shown that the ISGylation of the TSG101 protein by ISG15 controls EV secretion by promoting MVB targeting to the lysosome. Furthermore, ISGylation results in aggregation of the conjugated TSG101, which appears to act as a signal to propel MVBs towards fusion with lysosomes [50]. In contrast, EV secretion is influenced by actin cytoskeleton regulation, as cortactin knockout models have been shown to correlate with decreased EV secretion, while overexpression resulted in increased secretion [51].

Owing to their diverse content and ability to fuse with and release within other cells their shuttled molecular cargo, EVs are important mediators of the dynamic intercellular communication network. The molecular cargo (nucleic acids, proteins, and metabolites) delivered by EVs to recipient cells can alter their functional state and/or biological responses [20]. Due to the heterogeneity of their COO-dictated molecular cargo, EVs can have a diverse range of functional effects on their recipient cells. They have been shown to impact immune responses, viral pathogenicity, carcinogenesis, and the evolution of the tumor microenvironment.

## 3. The Impact of HPV-TEVs on the Tumor Microenvironment

There is increasing evidence that EVs released by malignant cells are involved in intercellular communication and can induce functional alterations in recipient cellular populations within the TME and at distant sites [34,52,53]. The biologically active cargo in EVs is generally transferred to recipient cells through the fusion of plasma membranes, receptor-mediated endocytosis, or phagocytosis [20,54]. Paradoxically, tumor-derived EVs (TEVs) can be immunosuppressive and immunostimulatory, with both types of signals being delivered simultaneously [55,56]. However, it is still unclear how multiple counterintuitive signals translate to functional inhibition or stimulation of the recipient cells [57,58,59]. TEVs have been shown to have an impact on neoplasia, tumor growth and metastasis, evolution of the TME, and resistance to therapy [20]. The TME is comprised of a complex milieu of stroma that includes blood and lymphatic endothelial cells, fibroblasts, mesenchymal cells, and pericytes. The TME also includes various innate and adaptive immune cell populations that can heavily influence the evolution of the TME in a protumorigenic or antitumorigenic manner [60,61]. TEVs released by HNC cells have been recently shown to functionally reprogram different cell types in the TME, including endothelial cells, macrophages, dendritic cells, T cells, B cells, and neurons [54,62,63,64,65,66,67,68,69]. Notably, whether TEVs from HPV-infected malignant cells play a major role in shaping the distinct HPV^+^ TME is an area of current investigation.

In several studies, HPV-TEVs were shown to have different protein-based cargo compared to those from HPV^−^ tumors in the head and neck region, with protein profiles that resembled their respective parental tumors [33,34,35,36]. HPV-TEVs contained *E6*/*E7*, p16, and survivin, whereas both types of EVs contained immunomodulatory molecules—OX40, OX40L, HSP70, FasL, and TGF-β. Notably, in comparison to EVs released by HPV^−^ tumors, HPV-TEVs promoted dendritic cell (DC) maturation and sustained DC function, insinuating a potential role in antitumor immunity [65]. A recent in silico analysis validated the findings of those studies through the observation of distinct immune landscapes that were dependent on HPV status and correlated with gene signatures of vesiculation—a surrogate for EV biogenesis [34]. Specifically, they found that the EV gene signature in HPV^+^ tumors correlated with an increased abundance of regulatory T cells (Tregs) and a decreased abundance of CD4^+^ T cells, cancer-associated fibroblasts (CAFs), and myeloid-derived suppressor cells (MDSCs). These findings need further validation to comprehensively understand the differences in the immune landscape of viral- and carcinogen-driven HNCs; however, they provide insight into distinct EV-based effects that could contribute to the disparity in the TMEs between the two HNC subtypes.

HPV-TEVs isolated from in vitro HNC cell lines have been shown to be enriched with immune effector-related antigens, such as CD47 and CD276, that are not present in TEVs from HPV^−^ HNC cell lines [70]. This is further evidence that TEVs isolated from HPV^+^ and HPV^−^ cell lines have different protein cargos, which may explain the differences in the immune landscape of these anatomically similar tumors and the observed disparity in their responses to treatment. Indeed, patients with HPV^+^ HNCs are more responsive to radiotherapy compared to their HPV^−^ counterparts [71,72,73]. There is compelling evidence that the reason for the superior radiosensitivity observed in patients with HPV^+^ HNCs is due to the presence of immune cells within the TME [74]. In a study conducted by Tong et al., the authors observed that HPV-TEVs derived from HPV^+^ HNCs were able to polarize macrophages into the antitumor M1 phenotype [75]. Furthermore, they found that this HPV-TEV-specific polarization increased the radiosensitivity of HNC cells. They attribute this functional modulation of macrophages to miR-9, which is a microRNA (miRNA) that was enriched in HPV-TEVs.

A recent study by Wang et al., utilizing 111 clinical samples, showed that EVs secreted from HPV^+^ HNC cells were enriched with the miRNA miR-9-5p—the miRNA most often associated with HPV [76]. Furthermore, they provide evidence that the transfer of miR-9-5p to CAFs was mediated by HPV-TEVs. This transfer resulted in the inhibition of TGF-β signaling that is implicated in the phenotypic transformation of fibroblasts through NOX4—an isoform of the NADPH oxidase enzymes (NOX) family that is ubiquitously expressed and continuously produces hydrogen peroxide [76,77]. In addition, lower levels of CAFs in the TME were associated with increased survival in patients with HNC. They conclude that miR-9-5p is associated with CAF infiltration in HPV^+^ HNCs and that a high level of miR-9-5p is correlated with a higher rate of patient survival [76].

In the cervix, the TME has been shown to be a major factor in the progression of cervical cancer (CC) [78]. An important hallmark of the transition from cervical precancerous lesions—cervical intraepithelial neoplasia 2 (CIN2)—to cancer and subsequent metastasis is the activation of proangiogenic genes and neo-vascularization [79,80]. The oncoproteins of HPV have been shown to directly induce the transcription of vascular endothelial growth factor (VEGF), a signal protein that stimulates the formation of blood vessels [81,82]. Notably, HPV-TEVs derived from HPV^+^ cervical cancer cell lines contained proangiogenic factors and had higher angiogenic potential compared to TEVs from HPV^−^ cervical carcinomas [83]. Furthermore, the underlying mechanism behind the enhanced angiogenic potential was attributed to the induction of key modulators of angiogenesis, VEGF-A, VEGFR-2, and angiopoietin-2, which are controlled by the hedgehog signaling pathway [81]. In addition, HPV-TEVs were taken up more readily by endothelial cells compared to their HPV^−^ counterparts [83].

HPV-TEVs secreted from HPV18^+^ HeLa cells were found to have a distinct miRNA signature upon silencing of the integrated HPV oncogenes *E6* and *E7* [84]. The identified signature included downregulated miRNAs let-7d-5p, miR-20a-5p, miR-378a-3p, miR-423-3p, miR-7-5p, and miR-92a-3 and upregulated miRNA miR-21-5p. These oncogene-dependent HPV-TEV miRNAs have been linked to the control of cell proliferation and apoptosis. Furthermore, in a study by Chiantore et al., miRNAs present in HPV-TEVs were analyzed in keratinocytes transduced with *E6* and *E7* from HPV16 [36]. They found that *E6* and/or *E7* can modify the expression of certain miRNAs that are typically involved in tumorigenesis, such as miR-18a, miR-19a, miR-34a, and miR-590-5. Moreover, HPV-TEVs were found to contain *E6* and *E7* mRNAs as cargo. In addition, another study detected the presence of HPV16 *E7* and other proteins, MUC16 and SIRPA, in circulating HPV-TEVs isolated from patients with aggressive oropharyngeal cancers [85]. Treatment of nontumorigenic epithelial cells with HPV-TEVs that harbored the aforementioned proteins increased invasion and induced epithelial-to-mesenchymal transition. These results highlight how the expression of HPV oncogenes affect the abundance of multiple miRNAs in TEVs that influence the growth of HPV^+^ cancer cells and potentiate the effects of the tumor virus on the surrounding environment.

An added layer of complexity to the TME is the presence of innervated tumors in some cancer types. Notably, innervated tumors have been found to be more aggressive than those that are less innervated [86,87,88,89]. Data from Madeo et al. showed that TEVs from HNC patients had neurite growth activity that was absent in EVs from healthy controls [66]. To determine whether TEV-mediated neurite outgrowth activity was associated with HPV status, they tested TEVs isolated from HPV^−^ and HPV^+^ cell lines on PC12 cells—rat pheochromocytoma cells extensively used in neurobiology that exhibit features of mature dopaminergic neurons [90]. Their results showed that TEVs from either HPV^+^ or HPV^−^ cell lines induced neurite outgrowth in vitro. However, TEVs isolated from HPV^−^ cell lines had significantly less activity compared to their HPV^+^ counterparts. Nevertheless, their results suggested that HPV status is not required for neurite outgrowth activity. Seeing as HPV-TEVs isolated from HPV^+^ cell lines had higher neurite outgrowth activity compared to their HPV^−^ counterparts, the authors extended their studies to CC—virtually all HPV^+^ [91]. In this subsequent study, their results showed that tumors isolated from CC patients were innervated in comparison to normal cervix tissue. Furthermore, they showed that HPV-TEVs from CC cell lines had neurite outgrowth activity. Taken together, HPV-TEVs isolated from both HNCs and CCs had neurite outgrowth activity, which promoted the innervation of these anatomically different tumors, highlighting the influence of HPV-TEVs in shaping the complex TME.

## 4. The Utility of HPV-TEVs in Liquid Biopsies

The utilization of EVs as liquid-based biomarkers of disease burden is being advanced for various cancers that include those associated with HPV infection [92,93]. Their presence in all biological fluids, multicomponent cargo that is reflective of their COO, and ease of sampling have the potential to provide “real-time” information on disease progression and/or response to therapy [27,93]. In a study by Tang et al., their group detected HPV16 DNA in 80% of EVs isolated from saliva of patients with HPV^+^ HNCs [94]. Notably, they observed elevated protein levels of six main glycolytic enzymes from isolated salivary EVs of patients with HPV^+^ HNC: aldolase (ALDOA), glyceraldehye-3-phosphate dehydrogenase (GAPDH), lactate dehydrogenase A/B (LDHA and LDHB), phosphoglycerate kinase 1 (PGK1), and pyruvate kinase M1/2 (PKM). In a recent study, our group has also identified alterations in various metabolic pathways that are associated with HPV status in HNCs [95]. This highlights how the molecular cargo of EVs contain similar constituents to those of the originating cell. In addition, the established protein signature from the saliva-based EVs was able to distinguish HPV^+^ HNCs from healthy controls [94]. 

In order to further elucidate the role of HPV-TEVs and their cargo in CC, RNA isolated from HPV-TEVs produced by HPV^+^ CC cell lines SiHa and HeLa was analyzed and compared to an HPV^−^ cell line C33a [35]. The results showed 3099 transcripts that were differentially enriched in HPV-TEVs compared to their HPV^−^ counterparts. Notably, among the top enriched transcripts were *EVC2*, *LUZP1*, and *ANKS1B*, which have been implicated in the hedgehog signaling pathway, cell migration, and invasion, respectively. Furthermore, Gene ontology (GO) and Kyoto Encyclopedia of Genes and Genomes (KEGG) analyses showed that the enriched transcripts found in HPV-TEVs played a role in axon guidance and tumor innervation. In addition, the HPV18 splice variant E6*I was consistently found in HPV-TEVs isolated from HeLa cells. This study suggests that the differential cargo present in HPV-TEVs, such as protumorigenic cellular RNA and viral transcripts, may have utility as clinical biomarkers of CC. These studies are prime examples of the differential multicomponent cargo present in EVs that are reflective of their COO, which allows, in this case, the ability to discriminate HPV^+^ carcinomas from healthy controls.

Currently, there are no biomarkers available for the early detection of oral cancers, and this often leads to their detection at an advanced stage that is associated with poor survival rates. When oral cancers are detected at an early stage, the 5-year survival rate increases from 40% to 80% [96,97]. Therefore, there is an unmet need for a reliable, painless, and noninvasive biomarker for early diagnoses and prognostication. In a recent study, the authors used RNA-seq to analyze EVs isolated from HPV^+^ hypopharynx cells (SCC2), HPV^+^ base-of-tongue cells (SCC90), and HPV^−^ tonsil cells (SCC72 and SCC89) [98]. Their results showed that 14 miRNAs were specifically enriched in HPV-TEVs isolated from HPV^+^ cell lines and 19 miRNAs specifically enriched in TEVs isolated from HPV^−^ cell lines. The enrichment of these miRNAs was specific to the HPV status of the COO and may provide utility as an EV-based biomarker for early detection and risk stratification in HNCs.

Recent evidence has shown that tumor cells actively release TEVs containing long-noncoding RNAs (lncRNAs) and that these molecules are differentially expressed in different cancers and have potential utility as diagnostic biomarkers [99,100]. In CC, the lncRNAs HOTAIR, MALAT1, and MEG3 were found to be enriched in HPV-TEVs isolated from cervicovaginal lavage samples [101]. Furthermore, the expression levels of the lncRNAs isolated from CC patients were significantly different compared to healthy controls. These results suggest that lncRNAs found in HPV-TEVs have potential as noninvasive biomarkers that could be used for the early detection and diagnosis of CC.

There are two main sources of DNA and/or RNA (DNA/RNA) that can be used for liquid biopsies. The first is cell-free (cf) DNA/RNA and the second is EV-associated DNA/RNA [102,103]. In a prospective study, the authors aimed to compare the detection sensitivity of both of these liquid biopsy sources to identify circulating HPV DNA/RNA in plasma isolated from patients with oropharyngeal squamous cell carcinomas (OSCC) [104]. Furthermore, they also analyzed longitudinal changes in HPV-DNA in response to treatment. Their results showed that circulating HPV-DNA was detected with higher sensitivity in cf-DNA compared to EV-associated DNA and similarly for HPV-RNA. In addition, 100% of the locally advanced cohort of HPV^+^ OSCC had a demonstrated reduction in the levels of circulating HPV-DNA following treatment, with 81% having levels that were undetectable. In contrast, patients with metastatic HPV^+^ OSCC had no correlation between HPV-DNA levels and treatment response. This study demonstrates that both sources of liquid biopsies can be used for the detection of HPV-DNA; however, it seems that cf-DNA/RNA is a more sensitive medium compared to EV-DNA/RNA. In addition, since HPV-DNA was only correlated to treatment response in the locally advanced cohort, the utility of HPV-DNA as a dynamic biomarker to assess treatment response requires further validation.

The cervical–vaginal fluid (CVF) has the potential to be used as a noninvasive medium for CC screening. This is due to the fact that EVs are broadly present in various body fluids and are representative of their COO. Several studies have compared the use of conventional cervical cytology with liquid-based cytology (LBC); some have found that conventional cervical cytology is more sensitive and specific as a diagnostic test compared to LBC [105,106], whereas others have found the opposite, that LBC was more sensitive and specific compared to conventional cervical cytology [107,108,109]. It is important to note that the LBCs used in these studies did not specifically isolate and use HPV-TEVs as their source of DNA and/or RNA.

In a recent study, EVs isolated from CVF from volunteers with or without an active HPV16 infection were used to explore the potential effects of miRNAs associated with HPV status on the development of CC [110]. Their results identified 45 and 55 miRNAs that were upregulated and downregulated, respectively, in response to infection with HPV16. GO and KEGG analyses revealed that the differentially expressed miRNAs were associated with tumorigenesis, specifically involved with the TGF-B, TNF, and Notch signaling pathways. This work highlights the differential cargo present in EVs associated with HPV infection compared to healthy volunteers that could potentially have utility as a noninvasive biomarker of disease.

Some HNCs, such as those of the oropharynx, are often late-stage diagnoses, which are associated with an increased mortality rate [111]. The reason for diagnoses at an advanced stage can be attributed to the nature of the cancer and the unique anatomical location, in that it often causes minimal symptoms and can metastasize to the local draining lymph nodes present in the neck [112]. Therefore, there is an unmet need to advance tools that can assist with the early detection of OSCC. A potential biomarker for early detection of HNCs are miRNAs; however, there is currently no clinically meaningful panel of miRNAs that can be used for such a feat. A study conducted by Mayne et al. aimed to develop a vigorous statistical approach to identify an EV-based miRNA signature to distinguish HPV^+^ OSCC patients from normal controls and those with gastroesophageal reflux disease (GERD), an inflammatory disease [113]. They isolated EVs from the serum of 20 control patients, 20 patients with GERD, and 40 patients with locally advanced HPV^+^ OSCC. Their results produced a regression model that contained 11 miRNA ratios that could be useful in the clinic. Furthermore, sample permutations indicated that the cross-validated prediction accuracy of the 11-miRNA ratio signature was not due to chance alone. This work clearly identified a panel of miRNAs isolated from EVs present in blood serum that was robustly cross-validated as a biomarker for detection of HPV^+^ OSCC. 

In another study [114], the mRNA cargo profiles of EVs isolated from HPV^+^ or HPV^−^ cell lines were distinct from one and other and resembled their COO. Indeed, paired cell lysates and EVs from HPV^+^ cells were enriched with *EGFR*, *TP53*, and *HSPA1A/B* mRNA, whereas those from HPV^−^ cells were enriched with *IL6*, *FAS*, and *DPP4* mRNA. Furthermore, they observed that miR-205-5p was exclusively present in HPV-TEVs, whereas miR-1972 was only detected in EVs from HPV^−^ cells. In addition, a study by Galiveti et al. aimed to evaluate the potential of miRNAs associated with HPV-TEVs from the head and neck region and determine their potential as a liquid biopsy for early detection of HNC [115]. Their results showed that the miRNAs present within HPV-TEVs were able to distinguish HNC cell lines from control oral epithelial cells. Furthermore, the EV-miRNA profiles were also different between early-stage HNC cases compared to cancer-free controls. These findings provide further evidence for the potential utility of miRNAs found in HPV-TEVs as biomarkers for HNCs.

## 5. Conclusions

The elucidation of the impact that HPV-TEVs have on infiltrating immune cells and/or nonmalignant cellular populations could help in unraveling the origins of the virally induced, immune-inflamed, HPV^+^ TME. Research initiatives that focus on viral cargo or altered cellular cargo enriched in HPV-TEVs that alter the biological responses of diverse cellular populations in the TME have the potential to help guide the rational design of therapies that can modulate and/or mobilize the local immune ecosystem towards an antitumor phenotype. In addition, the in-depth characterization of the multicomponent cargo of HPV-TEVs could be used in the development of EV-based liquid biopsies that are painless and provide “real-time” insights into disease burden.

Historically, DNA tumor viruses have been utilized as tools for studies that contributed to our understanding of molecular and cancer biology of mammalian cells [116,117]. Likewise, by studying how DNA tumor viruses hijack the EV biogenesis pathway in mammalian cells, we can exploit that knowledge to develop novel techniques of EV bioengineering to target cellular populations of interest and functionally reprogram them. Furthermore, knowledge can also be gained from these studies on novel methods for packaging molecular cargos, such as functional enzymes, therapeutic proteins, or antigens for delivery in the treatment of cancer, infectious diseases, or other debilitating disorders.

## Figures and Tables

**Table 1:** Global fraction of new cancers caused by HPV infection in 2018.

Site	New Cases (n)	Attributed to HPV (%)
Cervix	570,000	100
Oropharynx	42,000	30
Oral Cavity	280,000	2.1
Larynx	180,000	2.3
Anus	29,000	100
Penis	34,000	53
Vagina	18,000	78
Vulva	44,000	25
